# Cardiovascular Dysfunction and Altered Lysosomal Signaling in a Murine Model of Acid Sphingomyelinase Deficiency

**DOI:** 10.21203/rs.3.rs-5154105/v1

**Published:** 2025-03-20

**Authors:** Yun-Ting Wang, Alexandra K Moura, Rui Zuo, Kiana Roudbari, Jenny Z Hu, Saher A Khan, Zhengchao Wang, Yangping Shentu, Mi Wang, Pin-Lan Li, Jiukuan Hao, Yang Zhang, Xiang Li

**Affiliations:** University of Houston; University of Houston; University of Houston; University of Houston; University of Houston; University of Houston; University of Houston; Wenzhou Medical University; University of Houston; Virginia Commonwealth University; University of Houston; University of Houston; University of Houston

**Keywords:** Niemann-Pick Disease, acid sphingomyelinase deficiency, lysosome storage disorder, coronary microvascular dysfunction, cardiac pathology, pericyte-to-myofibroblast transition

## Abstract

Niemann-Pick Disease (NPD) is a rare autosomal recessive lysosomal storage disorder (LSD) caused by the deficiency of acid sphingomyelinase (ASMD), which is encoded by the *Smpd1* gene. ASMD impacts multiple organ systems in the body, including the cardiovascular system. This study is the first to characterize cardiac pathological changes in ASMD mice under baseline conditions, offering novel insights into the cardiac implications of NPD. Using histological analysis, biochemical assays, and echocardiography, we assessed cardiac pathological changes and function in *Smpd1*^−/−^ mice compared to *Smpd1*^+/+^ littermate controls. Immunofluorescence and biochemical assays demonstrated that ASMD induced lysosomal dysfunction, as evidenced by the accumulation of lysosomal-associated membrane proteins, lysosomal protease, and autophagosomes in pericytes and cardiomyocytes. This lysosomal dysfunction was accompanied by pericytes and cardiomyocytes inflammation, characterized by increased expression of caspase1 and inflammatory cytokines, and infiltration of inflammatory cells in the cardiac tissues of *Smpd1*^−/−^ mice. In addition, histological analysis revealed increased lipid deposition and cardiac steatosis, along with pericyte-to-myofibroblast transition (PMT) and interstitial fibrosis in *Smpd1*^−/−^ mice. Moreover, echocardiography further demonstrated that *Smpd1*^−/−^ mice developed coronary microvascular dysfunction (CMD), as evidenced by decreased coronary blood flow velocity and increased coronary arteriolar wall thickness. Additionally, these mice exhibited significant impairments in systolic and diastolic cardiac function, as shown by a reduced ejection fraction and prolonged left ventricular relaxation time constant (Tau value). These findings suggest that ASMD induces profound pathological changes and vascular dysfunction in the myocardium, potentially driven by mechanisms involving lysosomal dysfunction as well as both pericytes and cardiac inflammation.

## Introduction

Niemann-Pick disease (NPD) is a rare autosomal recessive disorder caused by acid sphingomyelinase (ASM) activity deficiency (ASMD) ^1^. ASM, encoded by the sphingomyelin phosphodiesterase 1 (Smpd1) gene, is a lysosomal hydrolase that breaks down sphingomyelin into phosphorylcholine and ceramide ^2^. Mutations in Smpd1 caused ASMD leads to the accumulation of sphingomyelin in lysosomes, resulting in lysosomal storage disorder (LSD) ^3^. The initial accumulation of lysosomal sphingomyelin in ASMD triggers the subsequent buildup of other lipids within lysosomes, with cholesterol being the most prominent ^4–6^. Furthermore, lipids derived from sphingomyelin, including ceramide and its downstream metabolites (e.g., sphingosine), lysosphingomyelin (sphingosylphosphorylcholine), glycosphingolipids, and bis(monoacylglycero)phosphate, also accumulate ^6^. Over time, these accumulated lipids are recycled to the plasma membrane and redistributed to other cellular compartments, disrupting cellular homeostasis and causing secondary abnormalities across multiple organelles ^6,7^. There are two subtypes of NPD caused by ASMD, NPD type A (NPD A) and NPD type B (NPD B). NPD A patients have less than 1% of normal enzyme activity, leading to the early onset of rapid systemic and neurodegenerative symptoms, typically reducing their lifespan to around 3 years ^8^. In contrast, NPD B patients have 5–10% of normal enzyme activity, do not exhibit neurodegenerative symptoms, and often survive into adulthood ^9^. However, these patients present with a highly variable clinical phenotype and disease-related morbidities affecting multiple organ systems, including respiratory and cardiovascular systems ^10^.

Cardiac disease accounts for 7.2% of deaths among NPD B patients, making it the fifth leading cause of mortality, following respiratory disease (27.7%), liver disease (27.7%), and bleeding (9.6%) ^10^. While NPD B patients have numerous cardiac conditions, valvular heart disease and coronary artery disease are the most common ^11^. Previous studies have shown that ASM activation and ceramide accumulation contribute to the development of cardiomyopathy and heart failure in conditions such as high-fat diet (HFD), myocardial infarction (MI), and sepsis ^12–16^. However, no comprehensive study has been conducted on the nature and extent of cardiac dysfunction and related cardiovascular events resulting from ASMD.

Proper sphingolipid homeostasis is essential for maintaining health, and disruption of this finely regulated balance can result in various diseases. This phenomenon is evident in several LSDs, such as Gaucher and Farber diseases, where inherited lysosomal defects lead to sphingolipid accumulation and subsequent pathogenesis ^17^. In this study, we use the Smpd1 knockout mouse model of ASMD to identify cardiac pathologies and dysfunction under baseline conditions. Our findings indicate that ASMD leads to perturbed cardiac function. ASMD-induced cardiac pathological changes encompass lysosomal dysfunction, cardiac inflammation, steatosis, fibrosis, coronary microvascular dysfunction (CMD), and overall cardiac dysfunction. These findings provide novel insights into the cardiac complications associated with ASMD.

## Materials and Methods

### Mice

All experimental protocols received approval from the University of Houston Institutional Animal Care and Use Committee. We used female wild-type (*Smpd1*^+/+^) and acid sphingomyelinase deficient (*Smpd1*^−/−^) mice, aged 5–6 months and of the C57BL/6J strain, for all experiments. The mice were kept in a temperature-controlled room with a 12-hour light-dark cycle and had free access to standard rodent chow and water. Following euthanasia, heart samples were collected and stored at −80°C for subsequent analysis.

### Echocardiography

Transthoracic echocardiography was conducted using a Vevo 3100 micro-ultrasound imaging system with an MX550D-0073 probe (VisualSonics Inc., Canada). Mice were anesthetized with 1.5–2% isoflurane (ISO) in 100% oxygen and maintained at 1–1.5% isoflurane throughout the procedure. M-mode images were acquired from the parasternal short-axis or apical four-chamber views and analyzed using Vevo LAB 2.1.0. The flow velocity of the left anterior descending coronary artery was measured in pulsed-wave Doppler mode under baseline conditions and after induction of hyperemia with 1.0% and 2.5% isoflurane, respectively. Left ventricular relaxation time constant (Tau value) = (IVRT + IVCT)/MV ET.

### Antibodies and reagents

Primary antibodies: NG2 (Abcam ab275024, Miltenyi Biotec 130–123-964), Troponin I (Abcam AB56357–1001), LAMP1 (BD 553792), LAMP2A (abcam ab18528), Cathepsin B (Abcam ab58802), LC3 (CST 12741S), p62 (Abcam ab109012), HMGB1 (Abcam ab79823), RAGE (Abcam ab3611), CD45 (Abcam ab25386), F4/80 (BD 565411), PLIN2 (Proteintech 15294–1-AP), TGFβ (CST 3711S), PDGFRa (CST 3174T, R&D AF1062-SP), FSP1 (CST 13018S), Pan-cadherin (Abcam ab51034), Vimentin (Abcam ab92547), Collagen 1 (Abcam ab21286), FITC-αSMA (Sigma F3777), Ceramide (Enzo, ALX-804–196-T050), β-actin (CST 3700S), GADPH (CST 2118S).

Secondary antibody for immunofluorescence: anti-Mouse IgG, Alexa Fluor 488 conjugate (Thermo Fisher A-21202), Alexa Fluor 488 conjugate anti-Rabbit IgG (Thermo Fisher A21206), Alexa Fluor 555 conjugate anti-Mouse IgG (Thermo Fisher A-31570), Alexa Fluor 555 conjugate anti-Rabbit IgG (Thermo Fisher A-31572). Secondary antibody for western blot: IRDye^®^ 800CW anti-Mouse IgG (LICOR 926–32212), IRDye^®^ 800CW anti-Rabbit IgG (LICOR 926–32213), anti-Mouse IgG, HRP (Thermo Fisher A16011), stabilized peroxidase conjugated anti-rabbit (Invitrogen 32460), anti-rat IgG-HRP (Fisher 629520).

Reagents: Oil red O (VWR BT135140–100G), Sirius red (Sigma 365548–5g), Isolectin GS-IB4 (Fisher I21411), WGA-FITC (Sigma L4895), Aurum Total RNA Mini Kits (Bio-Rad, 732–6820), iScript Reverse Transcription Supermix for RT-qPCR (Bio-Rad, 1708841), iTaq Universal SYBR Green Supermix (Bio-Rad, 1725121).

### Immunofluorescence staining

Frozen heart section slides were fixed with 4% paraformaldehyde (PFA) for 15 minutes at room temperature. After washing with PBS, the tissues were blocked and permeabilized using 5% BSA and 0.3% Triton X-100 in PBS for 1 hour at room temperature, then incubated with primary antibodies overnight at 4°C. Following washing with PBST (PBS with 0.05% Tween-20), the slides were incubated with secondary antibodies conjugated to Alexa Fluor 488 or Alexa Fluor 555 for 1 hour at room temperature. After another wash with PBST, the slides were mounted with DAPI mounting solution and analyzed using an Olympus IX73 or Leica STED 8 imaging system. Pearson correlation coefficient (PCC) of fluorescence image was quantified using Image-Pro Plus 6.0 software, as previously described ^18^. Frozen heart sections were stained with WGA-FITC (5 μg/ml in PBS) for 30 minutes at room temperature, followed by staining with Troponin I using the immunofluorescence staining protocol described above. The cardiomyocyte outlines were identified through Troponin I-positive staining and WGA-FITC labeling of cell membranes. Cardiomyocytes with centrally localized nuclei should be used for analysis to ensure consistent orthogonal cross-sectional measurements, minimizing the influence of obliquely oriented cardiomyocytes. Cardiomyocyte size was then measured using Image-Pro Plus 6.0 software.

### Hematoxylin and eosin (H&E) staining

H&E staining was carried out using a hematoxylin and eosin staining kit (Teomics HAE-1). Frozen heart sections were fixed in 4% paraformaldehyde (PFA) for 15 minutes at room temperature. After washing with distilled water, the tissues were stained with Mayer’s hematoxylin solution for 5 minutes, followed by two washes with distilled water to remove excess stain. A bluing reagent was then applied for 10–15 seconds, followed by two additional washes with distilled water. The slides were then dipped in absolute alcohol to remove excess moisture and stained with Eosin Y solution for 2–3 minutes. After rinsing with absolute alcohol, the slides were cleared and mounted in synthetic resin, followed by prompt imaging with the Olympus IX73 system.

### Oil Red O staining

Frozen heart sections were prepared at a thickness of 8 μm. The sections were air-dried on slides, fixed in 4% paraformaldehyde (PFA) for 15 minutes, and rinsed immediately in three changes of distilled water before being soaked for 20 minutes. To prevent water carryover, the sections were then washed with 60% isopropanol for 1 minute. Following staining with Oil Red O working solution for 30 minutes, the sections were rinsed with 60% isopropanol for 2 minutes and then rinsed twice with distilled water. Finally, the sections were mounted in mounting media, and images were captured promptly using the Olympus IX73 imaging system.

### Sirius red and fast green staining

Frozen heart section slides were fixed with Kahle fixative solution for 15 minutes at room temperature. After washing with PBS, the slides were stained with 0.1% Sirius Red and Fast Green in a saturated aqueous solution of picric acid at room temperature for 60 minutes. The slides were then washed in two changes of acidified water (0.1 N HCl in ddH2O) for 1 minute each. After a final wash with ddH2O, the sections were mounted in mounting media, and images were captured promptly using the Olympus IX73 imaging system.

### Quantitative Real-time PCR

Total RNA was isolated from heart tissue using Aurum Total RNA Mini Kits (Bio-Rad, 732–6820). cDNA was synthesized from the isolated RNA using iScript Reverse Transcription Supermix (Bio-Rad, 1708841). Real-time PCR was conducted with iTaq Universal SYBR Green Supermix (Bio-Rad, 1725121) on the Bio-Rad CFX Connect Real-Time System. Primers for *NIrp3*, *Asc*, *Caspase-1, IL1β, IL18, IL6, IL8*, *TNF, Icam1, and Vcam1* were obtained from Bio-Rad. Additional primers used in this study are listed in the Supplementary Material Table 1. Cycle threshold (Ct) values were converted to relative gene expression levels using the 2−ΔΔCt method, and the data were normalized to the internal controls β-actin or GAPDH.

### Western blotting

Heart tissue homogenates were lysed in Laemmli sample buffer (Bio-Rad, 161–0737), boiled at 95°C for 10 minutes, and then placed in an ice-cold ultrasonic bath for 5 minutes. The prepared samples were separated by 10–15% sodium dodecyl sulfate-polyacrylamide gels. Proteins were then transferred electrophoretically onto PVDF membranes at 35 V at 4°C overnight. The membranes were blocked with 5% BSA in Tris-buffered saline containing 0.05% Tween 20. After washing, the membranes were incubated with the appropriate primary antibodies, following the manufacturer’s instructions. Following incubation, the membranes were washed with PBST and subsequently incubated with corresponding secondary antibodies for 1 hour at room temperature. Finally, the bands were washed with PBST, visualized, and analyzed using the LI-COR^®^ Odyssey Fc System.

### Statistics analysis

Data are presented as mean ± standard error of the mean (SEM). Statistical comparisons between two groups were made using either a Student’s t-test or the Mann-Whitney test. All analyses were performed using GraphPad Prism 8.0 software (GraphPad Software, USA). A p-value of less than 0.05 was considered statistically significant.

## Results

### Lysosomal and autophagosome accumulation in cardiac pericyte and myocardium

**Figure 1:**
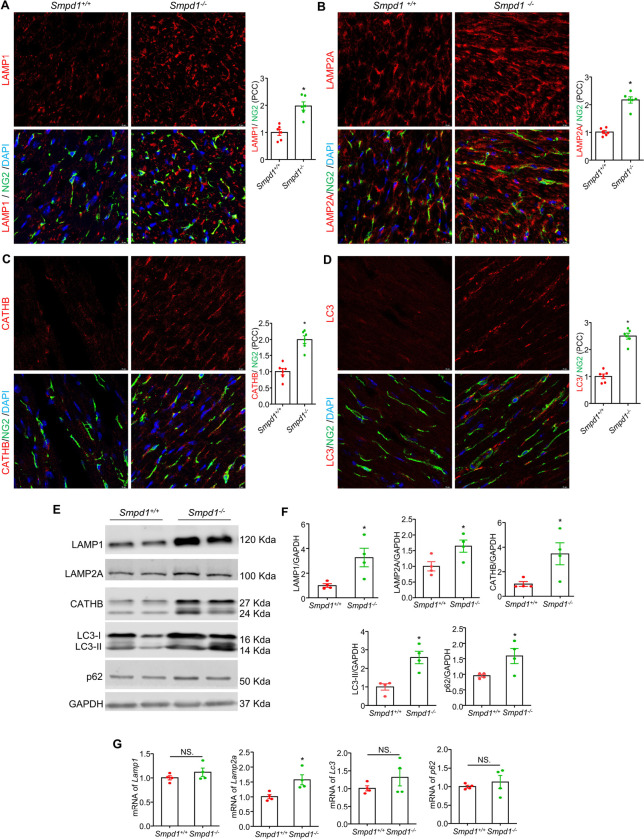
Lysosome components and autophagosome accumulation in pericyte and myocardium of ASMD mice. Representative immunofluorescence staining images for lysosomal-associated membrane protein LAMP1/NG2and summary of their PCC (**A**), LAMP2A/NG2 and summary of their PCC (**B**), lysosomal protease cathepsin B/NG2 and summary of their PCC(**C**), and autophagosome marker LC3/NG2 and summary of their PCC (**D**). NG2 is a marker for pericytes. DAPI stains nuclei. **E and F**, Representative immunoblots and summarized data for LAMP1, LAMP2A, Cathepsin B, LC3, and p62. **G,** mRNA levels of *Lamp1, Lamp2a, Lc3, and p62*. PCC, Pearson correlation coefficient; Scale bar =10 μm, *P< 0.05, NS. No Significance, (n=4–6).

Lysosomal storage disorder (LSD) is a hallmark and major contributor to lysosomal dysfunction in NPD ^19^. To investigate the presence of lysosomal dysfunction, we first performed immunofluorescence analyses of lysosome or autophagy-related proteins on cardiac sections focusing on cardiac pericyte and cardiomyocytes of *Smpd1*^−/−^ mice in comparison to *Smpd1*^+/+^ mice. The results showed lysosomal membrane protein LAMP1 (Fig. 1A) and LAMP2A (Fig. 1B), lysosomal protease cathepsin B (Fig. 1C), and the autophagosome membrane protein LC3 (Fig. 1D) were upregulated in cardiac pericyte of *Smpd1*^−/−^ mice. In addition to cardiac pericytes, these proteins were also elevated in the cardiomyocytes of Smpd1−/− mice (**Supplementary Fig. 1A-D**). These findings were further validated by immunoblot analyses (Fig. 1E, 1F). p62/Sqstm1, a macroautophagy receptor, is typically degraded during autophagy maturation, where autophagosomes fuse with lysosomes, leading to the degradation of their contents, including LC3 and p62/Sqstm1. Lysosomal dysfunction is often associated with impaired autophagic flux, resulting in the accumulation of LC3 and p62/Sqstm1. Consistent with the increased LC3 protein expression, the p62/Sqstm1 protein was also elevated in ASMD (Fig. 1E, 1F). However, the mRNA quantification of cardiac tissues showed that ASMD did not affect the gene expression of *Lamp1, Lc3*, and *p62*, but did lead to increased *Lamp2a* levels (Fig. 1G).

The mRNA quantification confirmed the absence of the *Smpd1* gene, but not the *Smpd2* gene, (encoding neutral sphingomyelinase/NSM) in the cardiac tissues of *Smpd1*^−/−^ mice (**Supplementary Fig. 2A**). Serine palmitoyltransferase (SPT), a multi-subunit enzyme, catalyzes the first and rate-limiting step in *de novo* ceramide synthesis ^20^. SPT activity is negatively regulated by ORMDL1–3, which are activated in response to increased ceramide levels ^21^. ASMD had no effect on gene expression of SPT subunit *Sptlc1* or *Ormdl1–3* in cardiac tissues but led to upregulation of *Sptlc2* (**Supplementary Fig. 2A**). Surprisingly, no significant difference in ceramide levels was observed between *Smpd1*^+/+^ and *Smpd1*^−/−^ mice in either cardiac pericytes (**Supplementary Fig. 2B**) or cardiomyocytes (**Supplementary Fig. 2C**). These results suggest that while ASMD significantly reduces *Smpd1* gene in cardiac tissues, it had compensatory upregulation of *Sptlc2*, but minimal impact on the NSM-dependent ceramide or other de novo ceramide synthesis pathways.

### Cardiac inflammation and cellular infiltration

**Figure 2:**
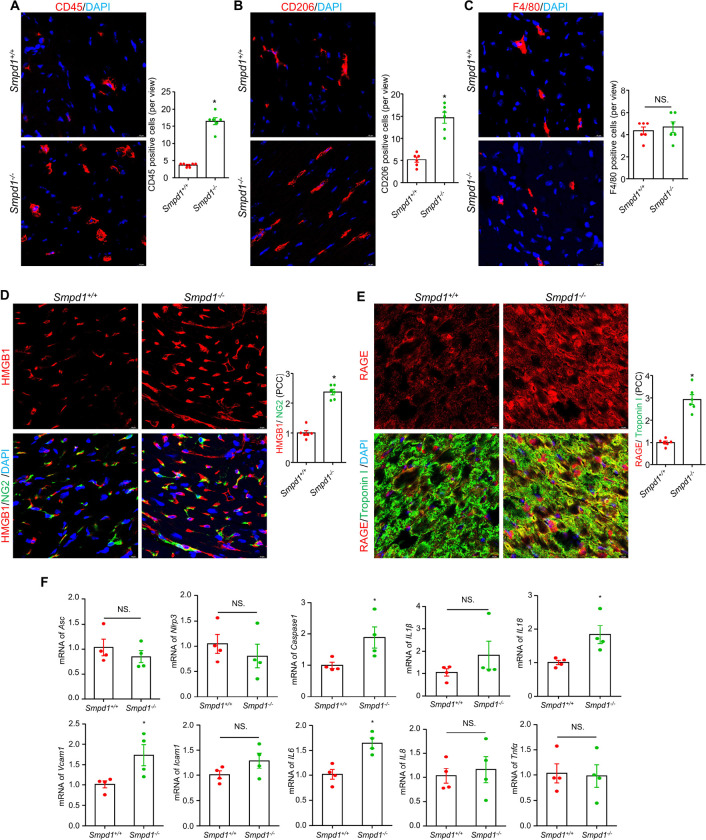
Cardiac inflammation and cellular infiltration in myocardium of ASMD mice. Cardiac inflammatory cells infiltration was indicated by CD45-positive leukocyte and summary of positive cell numbers per view **(A)**, CD206-positive macrophage and summary of positive cell numbers per view **(B)**, and F4/80-positive macrophage and summary of positive cell numbers per view**(C)**. Representative immunofluorescence staining images for inflammation markers: HMGB1/NG2 and summary of their PCC **(D)**, and RAGE/Troponin Iand summary of their PCC (**E**). NG2 is a marker for pericytes, Troponin I is a marker for cardiomyocytes. DAPI stains nuclei. **F,** mRNA levels of inflammasome and inflammation related genes: *Asc, NIrp3, Caspase-1, IL1β, IL18, IL6, IL8, Vcam1, Icam1, and Tnfα*. PCC, Pearson correlation coefficient; Scale bar =10 μm, *P< 0.05, NS. No Significance, (n=4–6).

Cardiac inflammation can lead to severe health conditions, including coronary artery disease, cardiac fibrosis, and heart failure ^22,23^. Immunofluorescence studies were first conducted on cardiac sections of *Smpd1*^−/−^ and *Smpd1*^+/+^ mice. The results revealed significantly increased infiltration of CD45-positive leukocytes (Fig. 2A) and CD206-positive macrophage (Fig. 2B) in the cardiac tissues in *Smpd1*^−/−^ mice compared to *Smpd1*^+/+^ mice. However, no increase in F4/80-positive macrophage infiltration was observed in *Smpd1*^−/−^ mice (Fig. 2C). To further characterize the inflammation in specific cell types, we analyzed the expression of HMGB1 (high mobility group box 1), a DAMP (damage-associated molecular pattern) linked to sterile inflammation, and its receptor RAGE (receptor for advanced glycation end-products) ^24^. We observed that ASMD-induced HMGB1 upregulation was primarily localized in non-cardiac cells, such as pericytes (Fig. 2D), while RAGE upregulation was predominantly observed in cardiomyocytes (Fig. 2E). In addition, mRNA quantification (Fig. 2F) from cardiac tissues of *Smpd1*^−/−^ mice showed increased expression of inflammation-related genes, including inflammatory *Caspase-1*, *Interleukin 18 (IL18), Vcam1* (vascular cell adhesion molecule 1), and *IL6*. However, no change was detected for other genes, including inflammasome component *Asc* (PYD and CARD domain-containing), *Nlrp3* (NLR family pyrin domain-containing 3), *IL1β, Icam1* (intercellular adhesion molecule 1), *IL8*, and *Tnfa*.

### Cardiac steatosis and lipid accumulation

**Figure 3:**
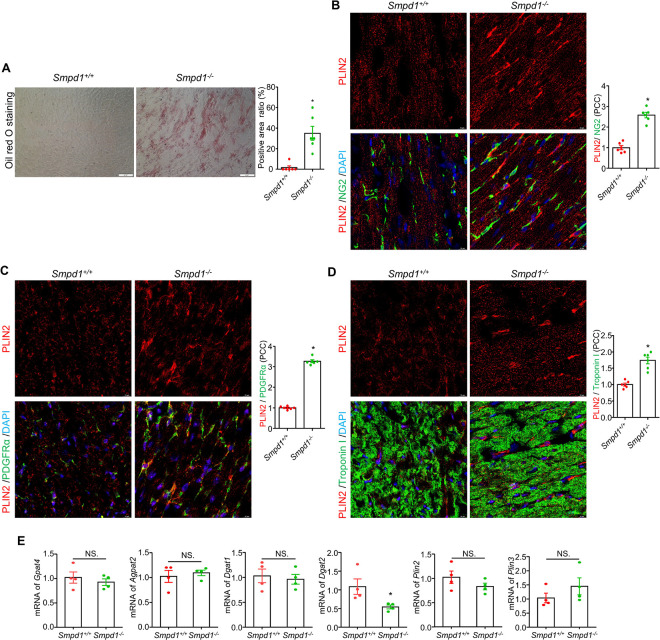
Cardiac steatosis and lipid accumulation in myocardium of ASMD mice. **A,** Representative Oil Red 0 staining for lipid deposition in cardiac tissues and summary ratio of Oil Red O positive areas over total view area under 40x lens. Representative immunofluorescence staining images for lipid droplets associated protein PLIN2/NG2 and summary of their PCC (**B**), PLIN2/PDGFRa and summary of their PCC (**C**), and PLIN2/Troponin I and summary of their PCC (**D**). NG2 is a marker for pericytes, PDGFRa is a marker for fibroblasts, Troponin I is a marker for cardiomyocytes. DAPI stains nuclei. **E,** mRNA levels of lipogenesis related genes: *Gpat4, Agpat2, Dgat1, Dgat2, Plin2, and Plin3*. PCC, Pearson correlation coefficient; Scale bar:20 μm for **A**, 10 μm for **B-D**, *P< 0.05, NS. No Significance, (n=4–6).

To investigate whether ASMD-induced lysosomal dysfunction is associated with altered lipid storage in the cardiac tissues, we performed Oil Red O staining and immunostained for the lipid droplet-associated protein perilipin 2 (PLIN2) in cardiac tissue sections from *Smpd1*^−/−^ and *Smpd1*^+/+^ mice. As shown in Fig. 3A, a significant increase in Oil Red O staining was observed in the cardiac tissues of *Smpd1*^−/−^ mice, whereas no positive staining was detected in *Smpd1*^+/+^ mice. Similarly, PLIN2 expression was significantly upregulated in *Smpd1*^−/−^ mice (Fig. 3B-D). We further confirmed that ASMD-induced PLIN2 accumulation was localized in cardiac pericytes (Fig. 3B), cardiac fibroblasts (Fig. 3C), and cardiomyocytes (Fig. 3D). We also examined the expression of genes involved in lipid droplet biogenesis, including *Gpat4* (glycerol-3-phosphate acyltransferase 4), *Agpat2* (1-acylglycerol-3-phosphate O-acyltransferase 2), *Dgat1/2* (diacylglycerol O-acyltransferase 1 and 2), and *Plin2/3*. As shown in Fig. 3E, the mRNA levels of most of these genes were comparable between *Smpd1*^−/−^ and *Smpd1*^+/+^ mice, except for a decrease in *Dgat2* expression. These findings suggest that ASMD leads to lipid deposition in the myocardium, resulting in cardiac steatosis, which is not associated with increased lipid droplet biogenesis.

### Cardiac fibrosis and pericyte-to-myofibroblast transition

**Figure 4:**
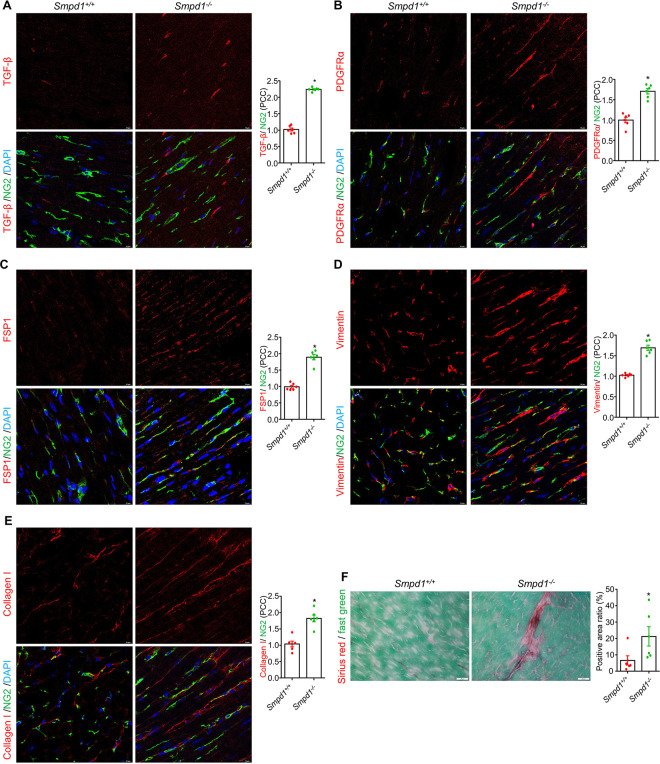
Cardiac fibrosis and pericyte-to-myofibroblast transition in myocardium of ASMD mice. **A-D,** Representative immunofluorescence staining images for pericyteto myofibroblast transition markers: TGF-β/NG2 and summary of their PCC (**A**), PDGFRa/NG2 and summary of their PCC (B), FSP1/NG2 and summary of their PCC (**C**), vimentin/NG2 and summary of their PCC (**D**), and collagen 1/NG2 and summary of their PCC (**E**). NG2 is a marker for pericytes. DAPI stains nuclei. Representative Sirius red and fast green staining images in cardiac interstitial, and their summary ratio of Sirius red staining positive areas over total view area under 40x lens (**F**). PCC, Pearson correlation coefficient; Scale bar:10 for **A-E**, 20 μm for **F**, *P< 0.05, NS. No Significance, (n=6).

While inflammation and steatosis were the most prominent cardiac changes observed in tissue samples from *Smpd1*^−/−^ mice, fibrosis was also evident. This was assessed using immunostaining for cardiac pericyte, fibroblast and myofibroblast markers, as well as Sirius red staining to visualize total collagen in cardiac tissue samples from *Smpd1*^−/−^ and *Smpd1*^+/+^ mice (Fig. 4A-F). Immunostaining demonstrated significant increases in the expression of proteins associated with pericyte-to-myofibroblast transition (PMT), a key process in cardiac fibrosis development ^25,26^. We demonstrated that ASMD increased transforming growth factor beta (TGF-β) (Fig. 4A), platelet-derived growth factor receptor alpha (PDGFRa) (Fig. 4B), fibroblast-specific protein 1 (FSP1) (Fig. 4C), vimentin (Fig. 4D) and collagen 1 (Fig. 4E) in cardiac pericytes. Sirius red staining confirmed an increase in collagen in the cardiac interstitial areas (Fig. 4F).

### Coronary microvascular dysfunction and arteriolar remodeling

**Figure 5:**
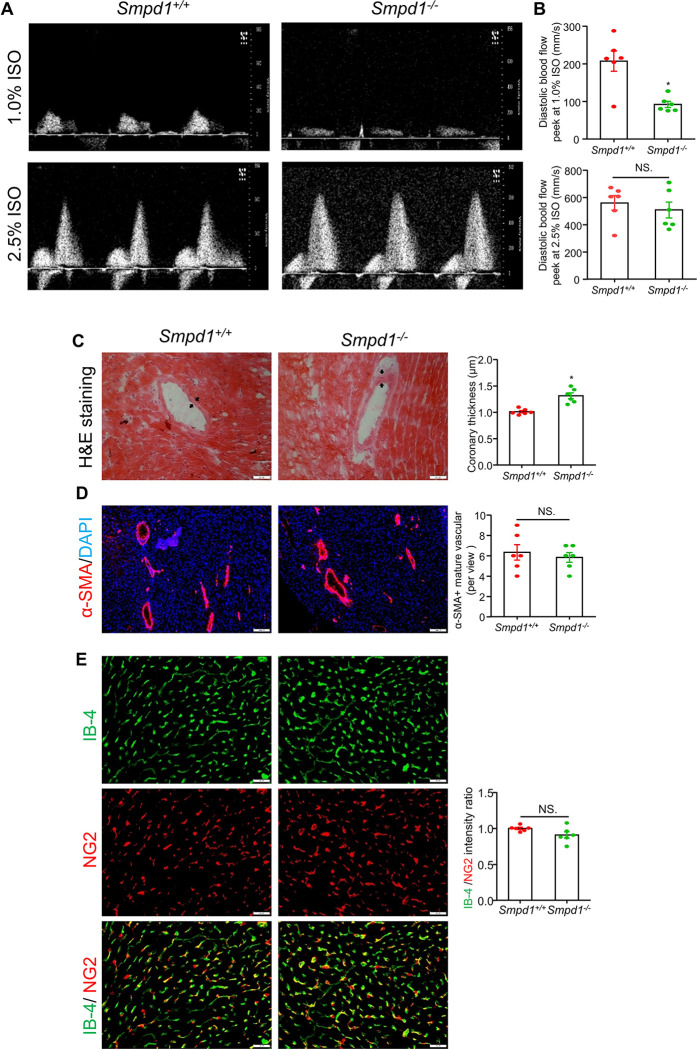
Coronary microvascular dysfunction and arteriolar remodeling in ASMD mice. **A,** Representative ultrasound images of isoflurane-induced vasodilation of the left anterior descending coronary artery at basal (1.0% ISO) and hyperemia (2.5% ISO) levels. **B,** Summarized data of diastolic blood flow velocity peeks at 1.0% ISO and 2.5% ISO. **C,** Representative H&E staining image and summary of coronary artery media thickness (black arrow). **D,** α-SMA-labeled mature coronary artery and number summary per view under 4x lens. **E,** Representative Isolectin-IB-4 labeled endothelial cells and NG2 labeled pericytes in capillary and summary in IB4/NG2 intensity ratio. DAPI stains nuclei. Scale bar:20 μm for **C** and **E**, 200 μm for **D**, *P< 0.05, NS. No Significance, (n=6).

Echocardiography was conducted to assess coronary microvascular function by measuring the coronary blood flow velocity of *Smpd1*^−/−^ and *Smpd1*^+/+^ mice at baseline conditions (1.0% isoflurane) as well as under hyperemic conditions (2.5% isoflurane). Representative images and summarized data (Fig. 5A and 5B) demonstrated a significant decrease in coronary blood flow velocity at baseline in *Smpd1*^−/−^ mice compared to *Smpd1*^+/+^ mice. However, no differences were observed under hyperemic conditions. Immunohistological analysis revealed increased coronary arteriolar wall thickness in *Smpd1*^−/−^ mice, which was attributed to increased media thickness, without evidence of neointima formation (Fig. 5C). Further immunofluorescence analysis was conducted to evaluate the density of coronary arterioles (Fig. 5D, α-SMA^+^ arterioles) and myocardial endothelial cells to pericytes coverage in capillaries (Fig. 5E, isolectin-IB4-labeled endothelial cells, NG2-labeled pericytes) in cardiac sections. These results showed no significant differences in the number of coronary arterioles and endothelial cells to pericytes coverage in capillaries between *Smpd1*^−/−^ and *Smpd1*^+/+^ mice. These findings suggest that ASMD leads to coronary arteriolar remodeling and impaired coronary microvascular function.

### Cardiac systolic and diastolic dysfunction

**Figure 6:**
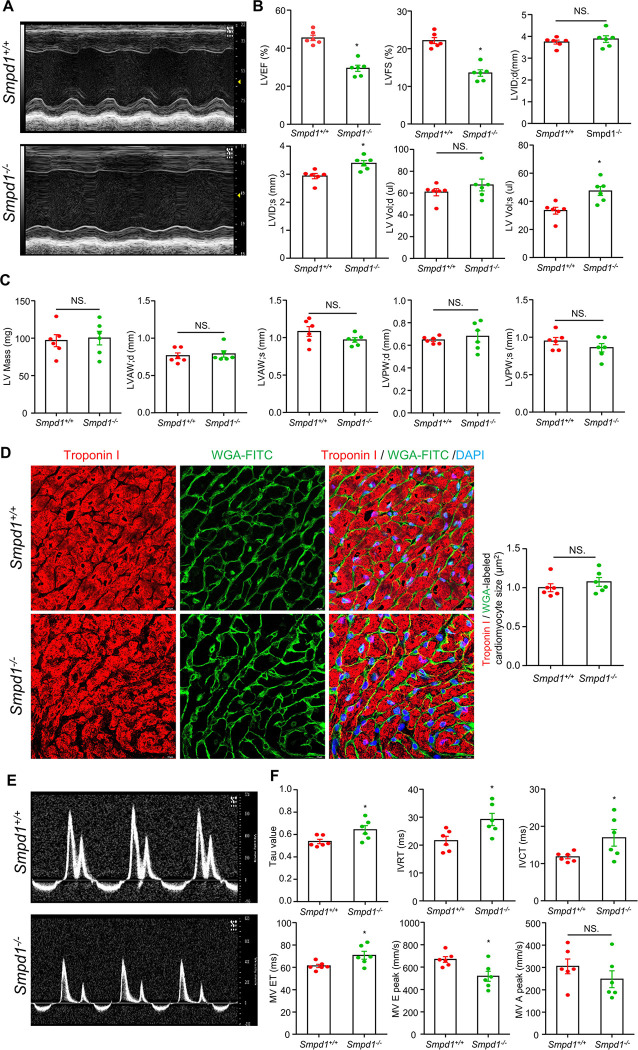
Cardiac systolic and diastolic dysfunction in ASMD mice. **A,** Representative M-mode images of left ventricular in parasternal short-axis view. **B,** Summarized data of echocardiographic parameters in cardiac systolic function: left ventricular ejection fraction (LVEF), left ventricular fractional shortening (LVFS), diastolic left ventricular internal end (LVID; d), systolic left ventricular internal end (LVID; s), diastolic left ventricle volume (LV Vol; d), and systolic left ventricle volume (LV Vol; s). **C,** Summarized data of echocardiographic parameters in cardiac remodeling: left ventricle mass (LV Mass), diastolic left ventricular anterior wall (LVAW; d), systolic left ventricular anterior wall (LVAW; s), diastolic left ventricular posterior wall (LVPW; d), and systolic left ventricular posterior wall (LVPW; s). **D,** Representative immunofluorescence staining images for Troponin I labeled cardiomyocytes and WGA-FITC labeled cell membrane, and summary in cardiomyocyte size. DAPI stains nuclei. **E,** Representative PW doppler mode images in apical four chamber view. **F,** Summarized data of echocardiographic parameters in cardiac diastolic function: Left ventricular relaxation time constant Tau value, isovolumic relaxation time (IVRT), isovolumic contraction time (IVCT), mitral valve ejection time (MV ET), MV E peak and MV A peak. Scale bar =10 μm, *P< 0.05, NS. No Significance, (n=6).

Finally, echocardiography was performed to investigate whether the cardiac pathologies and coronary microvascular dysfunction (CMD) observed in *Smpd1*^−/−^ mice are associated with changes in cardiac function and remodeling. Representative images and summarized data (Fig. 6A, 6B) revealed that *Smpd1*^−/−^ mice developed cardiac systolic dysfunction compared to *Smpd1*^+/+^ mice. Specifically, *Smpd1*^−/−^ mice exhibited reduced left ventricular ejection fraction (LVEF) and left ventricular fractional shortening (LVFS), along with increased systolic left ventricular internal diameter (LVID; s) and systolic left ventricle volume (LV Vol; s). However, there were no significant differences in diastolic left ventricular internal diameter (LVID; d) or diastolic left ventricle volume (LV Vol; d). As summarized in Fig. 6C, no significant cardiac remodeling was observed in *Smpd1*^−/−^ mice, as evidenced by the similar calculated left ventricular mass, diastolic left ventricular anterior wall thickness (LVAW; d), systolic left ventricular anterior wall thickness (LVAW; s), diastolic left ventricular posterior wall thickness (LVPW; d), and systolic left ventricular posterior wall thickness (LVPW; s) between the groups. In addition, there was no significant difference in cardiomyocytes size between *Smpd1*^+/+^ and *Smpd1*^−/−^ mice (Fig. 6D). Cardiac diastolic function of *Smpd1*^−/−^ and *Smpd1*^+/+^ mice was also assessed by measuring the left ventricular relaxation time constant (Tau value), isovolumic relaxation time (IVRT), isovolumic contraction time (IVCT), mitral valve ejection time (MVET), and mitral valve E/A (MV E/A) peak (Fig. 6E, 6F). The results indicated the development of cardiac diastolic dysfunction in *Smpd1*^−/−^ mice, as shown by increased IVRT, IVCT, and MV ET, alongside a reduction in MV E peak, with no change in MV A peak. Together, these results suggest that ASMD leads to heart failure characterized by both cardiac systolic and diastolic dysfunction.

## Discussion

The clinical manifestations of ASMD in patients with NPD are diverse and wide-ranging. In NPD A, the most frequent symptoms are central nervous system degeneration and respiratory failure. NPD B presents a broader range of clinical manifestations, with the most severely affected organs being the respiratory system, liver, and tissues associated with an atherogenic lipid profile ^9^. In addition, an increasing number of studies have reported cardiovascular diseases associated with NPD B ^3,9,10^. The *Smpd1* knockout mouse serve as a well-established murine model of NPD. In this study, we identified significant cardiac pathology in this model, showing that ASMD results in lysosomal dysfunction, cardiac inflammation, steatosis, fibrosis, CMD, and overall cardiac dysfunction.

LSDs encompass a group of diseases caused by deficiencies in lysosomal enzymes, membrane transporters, or other proteins critical for lysosomal function. These disorders are categorized based on the type of accumulated material, including lipids, mucopolysaccharidoses, and glycoproteinoses ^17,27^. ASM plays a key role in hydrolyzing sphingomyelin to ceramide within lysosomes, and in ASMD, this process is disrupted, leading to alterations in the ‘sphingolipid rheostat’ ^28^. This disruption results in imbalanced levels of sphingolipid species, such as sphingomyelin, ceramide, sphingosine, and their phosphorylated metabolites. These imbalances reorganize lysosomal membrane composition by forming sphingolipid-enriched microdomains ^29^, which in turn redistribute membrane-associated ion channels or proteins, influencing either their stability or function. Sphingolipids like sphingomyelin and sphingosine may also directly interact with lysosomal membrane ion channels, modulating their activities and contributing to lysosomal dysfunction. For example, the lysosomal membrane cation channel TRPML1 is primarily responsible for mediating lysosomal Ca ^2+^ release. Previous studies have shown that TRPML1-mediated lysosomal Ca ^2+^ release is markedly reduced in cells from NPD patients ^30^, likely due to the inhibitory effects of sphingomyelin accumulation within lysosomal membranes in ASMD. Our previous studies also confirmed that ASMD inhibits TRPML1-mediated lysosomal Ca^2+^ release, which impairs dynein-dependent autophagosome trafficking, resulting in defective lysosome-autophagosome fusion and diminished autophagic flux in vascular smooth muscle cells from *Smpd1*^−/−^ mice ^31–33^. These findings suggest that sphingolipids serve as major regulators for Ca^2+^-dependent lysosomal trafficking function and that ASMD disrupts this process, reducing the turnover of autophagosomes and lysosomes along with their contents. In the present study, we observed that ASMD increased the expression of lysosomal proteins LAMP1/2, cathepsin B, and autophagic proteins LC3 in cardiac pericytes and cardiomyocytes of *Smpd1*^−/−^ mice. Interestingly, ASMD had minimal effects on their mRNA levels, indicating that lysosome or autophagosome biogenesis is not substantially altered. Together, our findings reinforce the view that ASMD induces lysosomal dysfunction, leading to impaired autophagic flux in cardiac cells in a mouse model of NPD. Notably, there was no significant decrease in ceramide levels in ASMD mice in either cardiac pericytes or cardiomyocytes. Previous studies have reported higher ceramide levels in ASMD type B organoids compared to controls ^34^. This elevation in ceramide levels may result from the activity of other sphingomyelinases located in non-lysosomal compartments, as described in ASM knockout mice by other researchers ^35^. Furthermore, our data demonstrated an upregulation of the *Sptlc2* gene in ASMD mice (Supplementary Fig. 2A), which could enhance the de novo synthesis of ceramide, providing a compensatory mechanism. Sphingosine, a vital bioactive sphingolipid, is generated from ceramide through the enzymatic action of ceramidases and is subsequently phosphorylated by sphingosine kinases to produce sphingosine-1-phosphate (S1P), a molecule that promotes cell survival ^36^. The dynamic balance among sphingomyelin, ceramide, sphingosine, and S1P is crucial in modulating the effects of ASMD and determining the fate of the cell ^37,38^. Future research will aim to quantify sphingomyelin, ceramide, sphingosine, and S1P levels using lipidomics to clarify their roles in ASMD and their contributions to cellular dysfunction, including inflammation, lipid accumulation, and PMT. This strategy will address current limitations and provide critical insights into the sphingolipid-driven mechanisms underlying ASMD.

Accumulating evidence indicates that lysosomal dysfunction is intimately linked to inflammation through several mechanisms. Impaired autophagic flux caused by lysosomal dysfunction can lead to the accumulation of damaged organelles (e.g. damaged mitochondria), protein aggregates, and other undegraded cellular materials. In LSDs, severe dysfunction may cause lysosomal membrane destabilization or rupture which releases lysosomal enzymes and harmful substances, such as cathepsins, into the cytosol. These accumulated substances or lysosomal contents can be recognized by the immune system as DAMPs, triggering inflammatory responses through activation of pattern recognition receptors (PRRs). One of the best studied connections between the lysosomal-autophagy pathways and inflammation is the NLRP3 inflammasome pathway, which plays a key role in the progression of cardiovascular diseases ^22,39^. NLRP3 inflammasome activation leads to increased inflammatory caspase-1 activity and production of pro-inflammatory cytokines, such as IL-1β and IL-18. It can also activate inflammatory caspase-11 and induce GSDMD-dependent pore formation in the plasma membrane, causing pyroptotic cell death. Additionally, HMGB1 is increasingly recognized as a key DAMP molecule actively released by immune cells or passively released from dying cells during infection or sterile inflammation ^40^. HMGB1 is a multifunctional protein that exerts proinflammatory activity primarily by binding to RAGE ^24^. The HMGB1/RAGE axis is critical in the pathogenesis of inflammatory cardiomyopathy ^24,41^. Early inflammatory responses include the upregulation of adhesion molecules, such as VCAM-1 and ICAM-1, which facilitate the adhesion of CD45-positive leukocytes (e.g. lymphocytes, monocytes, eosinophils, and basophils) and CD206-positive macrophages to the myocardium ^42–44^. In this study, cardiac tissue from *Smpd1*^−/−^ mice showed a significant increase in the number of CD45-positive leukocytes, CD206-positive macrophages and protein expression of HMGB1 and RAGE, along with elevated expression of inflammation-related genes, including *Caspase1, IL18, Vcam1*, and *IL6*. These findings align with previous research showing that vascular smooth muscle cells from *Smpd1*^−/−^ mice exhibit higher gene expression of *IL18* and *IL6*, as well as increased monocyte adhesion at basal levels or after PDGF stimulation ^31^. Similarly, Elyse et al. reported that ASM knockdown in bronchial epithelial cells increased inflammation and neutrophil recruitment under both basal and infected conditions ^45^. In contrast, ASM was shown to mediate pro-atherogenic stimulus-induced inflammation in macrophages and endothelial cells ^46,47^. Furthermore, ASM activation can lead to excess ceramides, driving inflammation in various diseases, including cystic fibrosis (CF) ^48,49^. Therefore, both the loss of ASM activity leading to lysosomal dysfunction and the activation of ASM resulting in excess ceramide production can trigger inflammatory responses depending on the stimulus or disease context. Nonetheless, our findings provide evidence that lysosomal dysfunction is linked to cardiac inflammation in ASMD.

Cardiac steatosis refers to the abnormal accumulation of lipid droplets (mainly neutral triglycerides) within the myocardium. It has increasingly been recognized as a causative factor for cardiomyopathy, particularly in conditions such as severe aortic stenosis ^50^ and dilated cardiomyopathy ^51^. Selective overexpression of *DGAT1* (a key gene in lipogenesis) in cardiomyocytes induces cardiac steatosis and fibrosis ^52^, exacerbating angiotensin II-induced heart failure in mice ^53^. Although cardiac steatosis is often associated with metabolic conditions such as obesity, diabetes, and fatty liver disease, it can also occur in other pathological conditions, such as LSDs ^3^. In some LSDs, such as Gaucher disease and Farber disease, abnormal lipid metabolism results in lipid storage within cells, such as hepatocytes ^54^. Though not directly reported as a common symptom in LSDs, lipid droplet accumulation may occur in cardiomyocytes or other cell types in the myocardium and subsequently induce cardiac steatosis. Additionally, impaired autophagy caused by lysosomal dysfunction can exacerbate lipid accumulation by hindering the normal breakdown of lipid droplets. Indeed, in ASMD, cholesterol-enriched lipid droplets accumulate in human or murine macrophages, contributing to foam cell formation, a key mechanism in atherogenesis in metabolic disorders ^55^. However, the link between ASMD and cardiac steatosis has not previously been reported in murine models. In this study, we show for the first time that *Smpd1*^−/−^ mice exhibit a significant increase in cardiac steatosis without elevated expression of lipogenesis-related genes. This suggests that cardiac steatosis in ASMD is primarily due to impaired lipid droplet degradation rather than increased lipid droplet biogenesis.

Additionally, we observed the accumulation of the lipid droplet-associated protein PLIN2 not only in cardiomyocytes but also in cardiac pericytes and fibroblasts. The role of lipid droplets in the function of cardiac pericytes and fibroblasts remains largely unexplored and warrants further investigation. Our findings suggest that lipid droplets may play a pivotal role in the PMT process, which should be further studied in future research using *Smpd1* pericyte- or fibroblast-specific knockout mice.

Cardiac fibrosis, a complex pathophysiological process marked by excessive extracellular matrix accumulation, plays a key role in cardiac dysfunction and heart failure ^25^. A key cellular mechanism driving fibrosis is the pericyte-to-myofibroblast transition (PMT) ^25,26^. Fibrosis in various organs, such as the lung, liver, and endocardium, has been observed in patients with LSDs, including Farber disease and NPD B ^3,9^. In this study, *Smpd1*^−/−^ mice exhibited elevated expression of TGF-β in pericyte, increased PMT, and fibrotic myofibroblast markers such as FSP1, vimentin, and collagen 1 in pericyte, and increased interstitial collagen deposition in cardiac tissue. These findings suggest that ASMD may promote cardiac fibrosis through the activation of PMT. Recent studies have identified pericytes as a significant source of myofibroblast via PMT ^26,56^. Interestingly, our recent findings showed that vascular smooth muscle cells from *Smpd1*^−/−^ mice underwent a myofibrogenic transition when stimulated by PDGF, a process driven by lysosomal dysfunction, impaired autophagic flux, and the activation of p62/Nrf2 signaling axis ^31^. Since pericytes and vascular smooth muscle cells originate from the same perivascular progenitors, it is reasonable that ASMD in pericytes promotes PMT, contributing to cardiac fibrosis. M2 macrophages derived from monocytes are known to play a critical role in cardiac fibrosis, primarily by promoting the activation of myofibroblasts through the secretion of profibrotic factors such as TGF-β1 and IL-1β ^57,58^. Therefore, ASMD-induced cardiac fibrosis may result from the infiltration of CD206-positive M2 macrophages. Further studies are required to elucidate the mechanisms by which ASMD drives PMT, using pericyte-specific *Smpd1* knockout mouse models and cultured pericytes. In addition, previous studies have implicated ASM activation in fibrosis, suggesting that targeting ASM activity could be a therapeutic approach. For instance, imipramine or cardiomyocyte-specific *Smpd1* knockout has been shown to attenuate HFD-induced cardiac fibrosis via downregulating NADPH oxidase 4 (NOX4) ^12^. Similarly, astaxanthin has been found to reduce myocardial infarction-induced cardiac fibrosis by inhibiting ASM activity ^13^. Thus, like its role in cardiac inflammation, both ASMD and ASM overactivation can lead to fibrotic activation, depending on the specific stimulus or disease context. The findings from this study suggest that lysosomal dysfunction is closely associated with the development of cardiac fibrosis in ASMD.

Coronary microvascular dysfunction (CMD) refers to functional or structural abnormalities in the coronary microcirculation that lead to impaired myocardial perfusion ^59,60^. CMD can occur as a clinical condition independent of heart failure, and it may result in myocardial ischemia ^61,62^. This ischemia may manifest as symptoms such as chest pain (angina) and can contribute to the progression of heart failure over time. Previous studies have identified CMD in LSDs including Fabry disease ^63–66^ and Danon disease ^67^. In this study, non-invasive echocardiographic analysis of *Smpd1*^−/−^ mice revealed a significant reduction in coronary blood perfusion at baseline (1.0% isoflurane); however, no difference was observed under hyperemic conditions (2.5% isoflurane) (Fig. 5). Interestingly, this reduction at baseline was associated with structural remodeling of coronary arterioles, characterized by thickened arteriolar walls, while the number of mature coronary arterioles and endothelial cells to pericytes coverage in capillary remained unchanged (Fig. 5). Our findings of thickened coronary arteriolar walls align with previous studies showing coronary artery disease in NPD B ^11^, as well as increased inflammation and dedifferentiation of vascular smooth muscle cell from *Smpd1*^−/−^ mice ^31,33^. These findings support the view that ASMD promotes coronary arteriolar wall remodeling, resulting in CMD and impaired myocardial perfusion. Additionally, PMT and collagen 1 expression were increased in pericytes of interstitial capillary from *Smpd1*^−/−^ mice (Fig. 4), suggesting that extracellular matrix remodeling may increase capillary stiffness and exacerbate vasomotor dysfunction in thickened coronary arterioles. The precise mechanisms by which ASMD induces structural and functional changes in coronary microcirculation deserve further investigation.

**Figure 7:**
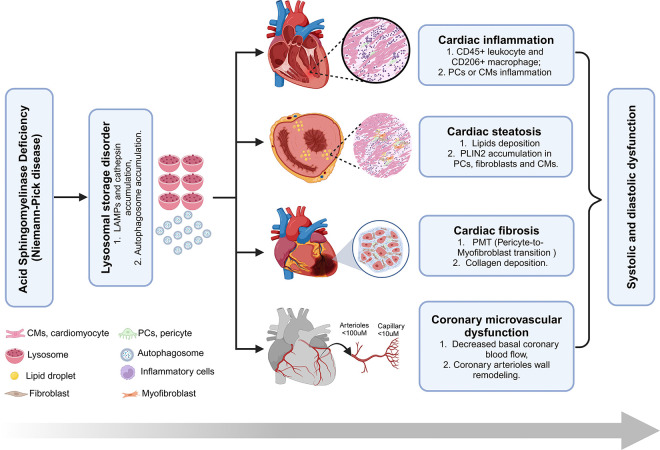
Proposed mechanisms by which ASM deficiency induces cardiovascular dysfunction in ASMD mice (see discussion for detail description).

Cardiac diseases, including hypertrophic and dilated cardiomyopathy, and valvular diseases, are commonly found in LSDs ^3^. Although cardiac disease accounts for 7.2% of deaths in NPD B patients, with a few reported cases of heart failure ^11,68,69^, cardiac dysfunction has not been extensively investigated in murine models of NPD or ASMD. In this study, non-invasive echocardiographic analysis of *Smpd1*^−/−^ mice showed both systolic and diastolic cardiac dysfunction under baseline conditions. Myocardial relaxation is one of the earliest manifestations of mechanical dysfunction of the human LV. The time constant tau (Ƭ) is higher in the elderly and patients with hypertrophic cardiomyopathy (HCM), coronary artery disease (CAD), and cardiomyopathies ^70^. Studies have shown that the lifespan of ASMD mice is typically less than 1 year, whereas wild-type mice generally live for over 2 years ^71,72^. Therefore, it is reasonable to consider the approximately 6-month-old ASMD mice in our study as aged mice, particularly since they exhibit upregulation of lysosomal accumulation, inflammation, and fibrosis–hallmarks commonly associated with aging ^73–75^. This ASMD-induced cardiac dysfunction was not associated with significant cardiac remodeling. Although ASMD induces significant cardiac fibrosis, it does not lead to notable cardiomyocyte hypertrophy. It would be valuable to investigate whether ASMD exacerbates cardiac remodeling in classical cardiac fibrosis models, such as isoproterenol-induced myocardial fibrosis. Our findings provide the first evidence linking ASMD to cardiac dysfunction in a murine model of NPD. While this study did not aim to further investigate the underlying mechanisms of ASMD and lysosomal dysfunction driving cardiac dysfunction, a lysosome-centered mechanistic model is proposed based on the observed cardiac pathologies (Fig. 7). In ASMD or NPD, the abnormal accumulation of lysosomal sphingomyelin leads to LSDs and lysosomal dysfunction in cardiac pericyte or cardiomyocyte. These lysosomal abnormalities may contribute to cardiac pericyte or cardiomyocyte inflammation/inflammasome activation, steatosis, PMT/fibrosis, and CMD. These pathological changes, either alone or in combination, ultimately lead to cardiac dysfunction. In future studies, we will implement targeted therapeutic strategies to address specific pathological changes in ASMD mice, aiming to identify the key contributors to ASMD-induced cardiac dysfunction.

Although our study provides significant evidence that ASMD induces a wide range of cardiac pathological changes, there are several limitations. First, the specific cell types involved in these pathological changes remain unclear and may vary depending on the context of the observed pathology. Future studies should explore the role of ASMD by employing tissue-specific *Smpd1* knock-out murine models in various cardiovascular cells, such as pericytes, cardiomyocytes, or fibroblasts, both under basal conditions and in disease models. This approach would provide a better understanding of how specific cell types contribute to the progression of ASMD-induced cardiac pathology. Second, although we propose a general mechanism by which ASMD-induced LSD leads to cardiac pathology, the lack of detailed mechanistic studies in the specific cell types limits the depth of our conclusions. Future *in vivo* or *in vitro* studies would provide definitive mechanistic insights into understanding the precise cellular and molecular pathways underlying ASMD-induced cardiac dysfunction.

In conclusion, the present study provides novel insights into the cardiac pathology in mice with ASMD. The most notable pathological changes reveal a complex interplay between lysosomal dysfunction, inflammation/inflammasome activation, lipid accumulation, cardiac fibrosis, CMD, and overall cardiac dysfunction. The profound ASMD-induced CMD and cardiac dysfunction observed in murine models suggest that similar mechanisms may contribute to cardiovascular complications in patients with NPD B or ASMD, highlighting the need for early diagnosis and intervention to improve patient outcomes. These findings lay the foundation for future research aimed at developing therapeutic targets for cardiovascular complications in patients with NPD B or ASMD, while also calling for further investigation into the complex factors contributing to ASMD-induced cardiovascular dysfunction.

## Data Availability

The authors declare that the data supporting the findings of this study are available within the paper, and its supplementary information files.

## Abbreviations

NPD: Niemann-Pick disease
NPD A: Niemann-Pick disease type A
NPD B: Niemann-Pick disease types B
LSD: Lysosome storage disorder
ASMase: Acid sphingomyelinase
ASMD: Acid sphingomyelinase deficiency
Smpd1: Sphingomyelin phosphodiesterase 1
Tau: Left ventricular relaxation time constant
ISO: Isoflurane
CMD: Coronary microvascular dysfunction
H&E: staining Hematoxylin and eosin staining
PFA: Paraformaldehyde
LVAW: Left ventricular anterior wall
LVID: Left ventricular internal end
LVPW: Left ventricular posterior wall
LVEF: Left ventricular ejection fraction
LVFS: Left ventricular shortening fraction
LV Vol: Left ventricle volume
IVRT: Isovolumic relaxation time
IVCT: Isovolumic contraction time
MV ET: Mitral valve ejection time
α-SMA: Alpha-smooth muscle actin
LD: Lipid droplet
PLIN2: Perilipin 2
GPAT4: Glycerol-3-phosphate acyltransferase 4
AGPAT2: 1-acylglycerol-3-phosphate O-acyltransferase 2
DGAT1: Diacylglycerol O-acyltransferase 1
DGAT2: Diacylglycerol O-acyltransferase 2
PMT: Pericyte-to-myofibroblast transition
TGF-β: Transforming growth factor beta
FSP1: Fibroblast-specific protein 1
HMGB1: High mobility group box 1
RAGE: Receptor for advanced glycation end-products
ASC: PYD and CARD domain containing
NLRP3: NLR family pyrin domain containing 3
IL1β: Interleukin 1 beta
IL18: Interleukin 18
VCAM1: Vascular cell adhesion molecule 1
ICAM1: Intercellular adhesion molecule 1
IL6: Interleukin 6
IL8: Interleukin 8
TNF-α: Tumor necrosis factor alpha
LAMP1: Lysosomal-associated membrane protein 1
LAMP2A: Lysosome-associated membrane protein type 2A
LC3A/B: Microtubule-associated proteins 1A/1B light chain 3A/B
p62: p62/SQSTM1
SPT: Serine palmitoyltransferase
SPTLC1: Serine palmitoyltransferase long chain base subunit 1
SPTLC2: Serine palmitoyltransferase long chain base subunit 2
ORMDL1: ORMDL Sphingolipid Biosynthesis Regulator 1
ORMDL2: ORMDL Sphingolipid Biosynthesis Regulator 2
ORMDL3: ORMDL Sphingolipid Biosynthesis Regulator 3
HFD: High fat diet
DAMP: Damage-associated molecular pattern
MI: Myocardial infarction
NG2: Nerve/glial antigen 2

## References

[1] Schuchman EH. The pathogenesis and treatment of acid sphingomyelinase-deficient Niemann-Pick disease. J Inherit Metab Dis. 2007;30:654–663. doi: 10.1007/s10545-007-0632-9 17632693

[2] Gorelik A, Illes K, Heinz LX, Superti-Furga G, Nagar B. Crystal structure of mammalian acid sphingomyelinase. Nat Commun. 2016;7:12196. doi: 10.1038/ncomms12196 27435900 PMC4961792

[3] Nair V, Belanger EC, Veinot JP. Lysosomal storage disorders affecting the heart: a review. Cardiovasc Pathol. 2019;39:12–24. doi: 10.1016/j.carpath.2018.11.002 30594732

[4] Walkley SU, Vanier MT. Secondary lipid accumulation in lysosomal disease. Biochim Biophys Acta. 2009;1793:726–736. doi: 10.1016/j.bbamcr.2008.11.014 19111580 PMC4382014

[5] Vanier MT. Biochemical studies in Niemann-Pick disease. I. Major sphingolipids of liver and spleen. Biochim Biophys Acta. 1983;750:178–184. doi: 10.1016/0005-2760(83)90218-7 6824712

[6] Breiden B, Sandhoff K. Mechanism of Secondary Ganglioside and Lipid Accumulation in Lysosomal Disease. Int J Mol Sci. 2020;21. doi: 10.3390/ijms21072566 32272755 PMC7178057

[7] Geberhiwot T, Wasserstein M, Wanninayake S, Bolton SC, Dardis A, Lehman A, Lidove O, Dawson C, Giugliani R, Imrie J, Consensus clinical management guidelines for acid sphingomyelinase deficiency (Niemann-Pick disease types A, B and A/B). Orphanet J Rare Dis. 2023;18:85. doi: 10.1186/s13023-023-02686-6 37069638 PMC10108815

[8] Ledesma MD, Prinetti A, Sonnino S, Schuchman EH. Brain pathology in Niemann Pick disease type A: insights from the acid sphingomyelinase knockout mice. J Neurochem. 2011;116:779–788. doi: 10.1111/j.1471-4159.2010.07034.x 21214563 PMC3059095

[9] McGovern MM, Avetisyan R, Sanson BJ, Lidove O. Disease manifestations and burden of illness in patients with acid sphingomyelinase deficiency (ASMD). Orphanet J Rare Dis. 2017;12:41. doi: 10.1186/s13023-017-0572-x 28228103 PMC5322625

[10] Cassiman D, Packman S, Bembi B, Turkia HB, Al-Sayed M, Schiff M, Imrie J, Mabe P, Takahashi T, Mengel KE, Cause of death in patients with chronic visceral and chronic neurovisceral acid sphingomyelinase deficiency (Niemann-Pick disease type B and B variant): Literature review and report of new cases. Mol Genet Metab. 2016;118:206–213. doi: 10.1016/j.ymgme.2016.05.001 27198631

[11] McGovern MM, Lippa N, Bagiella E, Schuchman EH, Desnick RJ, Wasserstein MP. Morbidity and mortality in type B Niemann-Pick disease. Genet Med. 2013;15:618–623. doi: 10.1038/gim.2013.4 23412609

[12] Liu R, Duan T, Yu L, Tang Y, Liu S, Wang C, Fang WJ. Acid sphingomyelinase promotes diabetic cardiomyopathy via NADPH oxidase 4 mediated apoptosis. Cardiovasc Diabetol. 2023;22:25. doi: 10.1186/s12933-023-01747-1 36732747 PMC9896821

[13] Shi Y, Lin P, Wang X, Zou G, Li K. Sphingomyelin phosphodiesterase 1 (SMPD1) mediates the attenuation of myocardial infarction-induced cardiac fibrosis by astaxanthin. Biochem Biophys Res Commun. 2018;503:637–643. doi: 10.1016/j.bbrc.2018.06.054 29906461

[14] Chung HY, Kollmey AS, Schrepper A, Kohl M, Blass MF, Stehr SN, Lupp A, Graler MH, Claus RA. Adjustment of Dysregulated Ceramide Metabolism in a Murine Model of Sepsis-Induced Cardiac Dysfunction. Int J Mol Sci. 2017;18. doi: 10.3390/ijms18040839 28420138 PMC5412423

[15] Kott M, Elke G, Reinicke M, Winoto-Morbach S, Schadler D, Zick G, Frerichs I, Weiler N, Schutze S. Acid sphingomyelinase serum activity predicts mortality in intensive care unit patients after systemic inflammation: a prospective cohort study. PLoS One. 2014;9:e112323. doi: 10.1371/journal.pone.0112323 25384060 PMC4226549

[16] Doehner W, Bunck AC, Rauchhaus M, von Haehling S, Brunkhorst FM, Cicoira M, Tschope C, Ponikowski P, Claus RA, Anker SD. Secretory sphingomyelinase is upregulated in chronic heart failure: a second messenger system of immune activation relates to body composition, muscular functional capacity, and peripheral blood flow. Eur Heart J. 2007;28:821–828. doi: 10.1093/eurheartj/ehl541 17353227

[17] Platt FM, d’Azzo A, Davidson BL, Neufeld EF, Tifft CJ. Lysosomal storage diseases. Nat Rev Dis Primers. 2018;4:27. doi: 10.1038/s41572-018-0025-4 30275469

[18] Wang YT, Li X, Chen J, McConnell BK, Chen L, Li PL, Chen Y, Zhang Y. Activation of TFEB ameliorates dedifferentiation of arterial smooth muscle cells and neointima formation in mice with high-fat diet. Cell Death Dis. 2019;10:676. doi: 10.1038/s41419-019-1931-4 31515484 PMC6742653

[19] Lan MY, Kang TW, Lan SC, Huang WT. Spontaneous splenic rupture as the first clinical manifestation of Niemann-Pick disease type B: A case report and review of the literature. J Clin Lipidol. 2022;16:434–437. doi: 10.1016/j.jacl.2022.06.002 35988956

[20] Lone MA, Hulsmeier AJ, Saied EM, Karsai G, Arenz C, von Eckardstein A, Hornemann T. Subunit composition of the mammalian serine-palmitoyltransferase defines the spectrum of straight and methyl-branched long-chain bases. Proc Natl Acad Sci U S A. 2020;117:15591–15598. doi: 10.1073/pnas.2002391117 32576697 PMC7355037

[21] Xie T, Liu P, Wu X, Dong F, Zhang Z, Yue J, Mahawar U, Farooq F, Vohra H, Fang Q, Ceramide sensing by human SPT-ORMDL complex for establishing sphingolipid homeostasis. Nat Commun. 2023;14:3475. doi: 10.1038/s41467-023-39274-y 37308477 PMC10261145

[22] Zheng Y, Xu L, Dong N, Li F. NLRP3 inflammasome: The rising star in cardiovascular diseases. Front Cardiovasc Med. 2022;9:927061. doi: 10.3389/fcvm.2022.927061 36204568 PMC9530053

[23] Thomas TP, Grisanti LA. The Dynamic Interplay Between Cardiac Inflammation and Fibrosis. Front Physiol. 2020;11:529075. doi: 10.3389/fphys.2020.529075 33041853 PMC7522448

[24] Bangert A, Andrassy M, Muller AM, Bockstahler M, Fischer A, Volz CH, Leib C, Goser S, Korkmaz-Icoz S, Zittrich S, Critical role of RAGE and HMGB1 in inflammatory heart disease. Proc Natl Acad Sci U S A. 2016;113:E155–164. doi: 10.1073/pnas.1522288113 26715748 PMC4720305

[25] Hoque MM, Gbadegoye JO, Hassan FO, Raafat A, Lebeche D. Cardiac fibrogenesis: an immuno-metabolic perspective. Front Physiol. 2024;15:1336551. doi: 10.3389/fphys.2024.1336551 38577624 PMC10993884

[26] Alex L, Tuleta I, Hernandez SC, Hanna A, Venugopal H, Astorkia M, Humeres C, Kubota A, Su K, Zheng D, Cardiac Pericytes Acquire a Fibrogenic Phenotype and Contribute to Vascular Maturation After Myocardial Infarction. Circulation. 2023;148:882–898. doi: 10.1161/CIRCULATIONAHA.123.064155 37350296 PMC10527624

[27] Zuo R, Wang M, Wang YT, ShenTu Y, Moura AK, Zhou Y, Roudbari K, Hu JZ, Li PL, Hao J, Ablation of Hepatic Asah1 Gene Disrupts Hepatic Lipid Homeostasis and Promotes Fibrotic Nonalcoholic Steatohepatitis in Mice. Am J Pathol. 2024. doi: 10.1016/j.ajpath.2024.11.003 PMC11983695 39719015

[28] Breiden B, Sandhoff K. Acid Sphingomyelinase, a Lysosomal and Secretory Phospholipase C, Is Key for Cellular Phospholipid Catabolism. Int J Mol Sci. 2021;22. doi: 10.3390/ijms22169001 34445706 PMC8396676

[29] Zhang Y, Li X, Becker KA, Gulbins E. Ceramide-enriched membrane domains--structure and function. Biochim Biophys Acta. 2009;1788:178–183. doi: 10.1016/j.bbamem.2008.07.030 18786504

[30] Medina DL. TRPML1 and TFEB, an Intimate Affair. Handb Exp Pharmacol. 2023;278:109–126. doi: 10.1007/164_2022_603 35879578

[31] Zhang P, Guan Y, Chen J, Li X, McConnell BK, Zhou W, Boini KM, Zhang Y. Contribution of p62/SQSTM1 to PDGF-BB-induced myofibroblast-like phenotypic transition in vascular smooth muscle cells lacking Smpd1 gene. Cell Death Dis. 2018;9:1145. doi: 10.1038/s41419-018-1197-2 30451833 PMC6242941

[32] Xu M, Zhang Q, Li PL, Nguyen T, Li X, Zhang Y. Regulation of dynein-mediated autophagosomes trafficking by ASM in CASMCs. Front Biosci (Landmark Ed). 2016;21:696–706. doi: 10.2741/4415 26709800 PMC4703364

[33] Li X, Xu M, Pitzer AL, Xia M, Boini KM, Li PL, Zhang Y. Control of autophagy maturation by acid sphingomyelinase in mouse coronary arterial smooth muscle cells: protective role in atherosclerosis. J Mol Med (Berl). 2014;92:473–485. doi: 10.1007/s00109-014-1120-y 24463558 PMC4211081

[34] Gomez-Mariano G, Perez-Luz S, Ramos-Del Saz S, Matamala N, Hernandez-SanMiguel E, Fernandez-Prieto M, Gil-Martin S, Justo I, Marcacuzco A, Martinez-Delgado B. Acid Sphingomyelinase Deficiency Type B Patient-Derived Liver Organoids Reveals Altered Lysosomal Gene Expression and Lipid Homeostasis. Int J Mol Sci. 2023;24. doi: 10.3390/ijms241612645 37628828 PMC10454326

[35] Schuchman EH, Wasserstein MP. Types A and B Niemann-Pick disease. Best Pract Res Clin Endocrinol Metab. 2015;29:237–247. doi: 10.1016/j.beem.2014.10.002 25987176

[36] Olivera A, Kohama T, Edsall L, Nava V, Cuvillier O, Poulton S, Spiegel S. Sphingosine kinase expression increases intracellular sphingosine-1-phosphate and promotes cell growth and survival. J Cell Biol. 1999;147:545–558. doi: 10.1083/jcb.147.3.545 10545499 PMC2151183

[37] Smith EL, Schuchman EH. The unexpected role of acid sphingomyelinase in cell death and the pathophysiology of common diseases. FASEB J. 2008;22:3419–3431. doi: 10.1096/fj.08-108043 18567738 PMC2537423

[38] Pyne S, Pyne NJ. Sphingosine 1-phosphate signalling in mammalian cells. Biochem J. 2000;349:385–402. doi: 10.1042/0264-6021:3490385 10880336 PMC1221160

[39] Halade GV, Lee DH. Inflammation and resolution signaling in cardiac repair and heart failure. EBioMedicine. 2022;79:103992. doi: 10.1016/j.ebiom.2022.103992 35405389 PMC9014358

[40] Andersson U, Tracey KJ. HMGB1 is a therapeutic target for sterile inflammation and infection. Annu Rev Immunol. 2011;29:139–162. doi: 10.1146/annurev-immunol-030409-101323 21219181 PMC4536551

[41] Tan H, Hu J, Zuo W, Huang Y, Cui J, Gong F, Bai W. Activation of the High Mobility Group Box 1/Receptor for Advanced Glycation Endproducts /NOD-like Receptor Family Pyrin Domain-Containing 3 Axis Under Chronic Intermittent Hypoxia Induction Promotes the Progression of Atherosclerosis in ApoE(−/−) Mice. J Am Heart Assoc. 2023;12:e024397. doi: 10.1161/JAHA.121.024397 37026550 PMC10227261

[42] Martini E, Kunderfranco P, Peano C, Carullo P, Cremonesi M, Schorn T, Carriero R, Termanini A, Colombo FS, Jachetti E, Single-Cell Sequencing of Mouse Heart Immune Infiltrate in Pressure Overload-Driven Heart Failure Reveals Extent of Immune Activation. Circulation. 2019;140:2089–2107. doi: 10.1161/CIRCULATIONAHA.119.041694 31661975

[43] Antipenko S, Mayfield N, Jinno M, Gunzer M, Ismahil MA, Hamid T, Prabhu SD, Rokosh G. Neutrophils are indispensable for adverse cardiac remodeling in heart failure. J Mol Cell Cardiol. 2024;189:1–11. doi: 10.1016/j.yjmcc.2024.02.005 38387309 PMC10997476

[44] Rheinlander A, Schraven B, Bommhardt U. CD45 in human physiology and clinical medicine. Immunol Lett. 2018;196:22–32. doi: 10.1016/j.imlet.2018.01.009 29366662

[45] MacFadden-Murphy E, Roussel L, Martel G, Berube J, Rousseau S. Decreasing SMPD1 activity in BEAS-2B bronchial airway epithelial cells results in increased NRF2 activity, cytokine synthesis and neutrophil recruitment. Biochem Biophys Res Commun. 2017;482:645–650. doi: 10.1016/j.bbrc.2016.11.087 27865842

[46] Jin J, Zhang X, Lu Z, Perry DM, Li Y, Russo SB, Cowart LA, Hannun YA, Huang Y. Acid sphingomyelinase plays a key role in palmitic acid-amplified inflammatory signaling triggered by lipopolysaccharide at low concentrations in macrophages. Am J Physiol Endocrinol Metab. 2013;305:E853–867. doi: 10.1152/ajpendo.00251.2013 23921144 PMC3798699

[47] Koka S, Xia M, Chen Y, Bhat OM, Yuan X, Boini KM, Li PL. Endothelial NLRP3 inflammasome activation and arterial neointima formation associated with acid sphingomyelinase during hypercholesterolemia. Redox Biol. 2017;13:336–344. doi: 10.1016/j.redox.2017.06.004 28633109 PMC5479959

[48] Seitz AP, Grassme H, Edwards MJ, Pewzner-Jung Y, Gulbins E. Ceramide and sphingosine in pulmonary infections. Biol Chem. 2015;396:611–620. doi: 10.1515/hsz-2014-0285 25720061

[49] Zhang Y, Li X, Carpinteiro A, Gulbins E. Acid sphingomyelinase amplifies redox signaling in Pseudomonas aeruginosa-induced macrophage apoptosis. J Immunol. 2008;181:4247–4254. doi: 10.4049/jimmunol.181.6.4247 18768882

[50] Mahmod M, Bull S, Suttie JJ, Pal N, Holloway C, Dass S, Myerson SG, Schneider JE, De Silva R, Petrou M, Myocardial steatosis and left ventricular contractile dysfunction in patients with severe aortic stenosis. Circ Cardiovasc Imaging. 2013;6:808–816. doi: 10.1161/CIRCIMAGING.113.000559 23833283

[51] Graner M, Pentikainen MO, Nyman K, Siren R, Lundbom J, Hakkarainen A, Lauerma K, Lundbom N, Nieminen MS, Petzold M, Cardiac steatosis in patients with dilated cardiomyopathy. Heart. 2014;100:1107–1112. doi: 10.1136/heartjnl-2013-304961 24763492

[52] Glenn DJ, Wang F, Nishimoto M, Cruz MC, Uchida Y, Holleran WM, Zhang Y, Yeghiazarians Y, Gardner DG. A murine model of isolated cardiac steatosis leads to cardiomyopathy. Hypertension. 2011;57:216–222. doi: 10.1161/HYPERTENSIONAHA.110.160655 21220706 PMC3322545

[53] Glenn DJ, Cardema MC, Ni W, Zhang Y, Yeghiazarians Y, Grapov D, Fiehn O, Gardner DG. Cardiac steatosis potentiates angiotensin II effects in the heart. Am J Physiol Heart Circ Physiol. 2015;308:H339–350. doi: 10.1152/ajpheart.00742.2014 25485904 PMC4329483

[54] Yu FPS, Molino S, Sikora J, Rasmussen S, Rybova J, Tate E, Geurts AM, Turner PV, McKillop WM, Medin JA. Hepatic pathology and altered gene transcription in a murine model of acid ceramidase deficiency. Lab Invest. 2019;99:1572–1592. doi: 10.1038/s41374-019-0271-4 31186526

[55] Wallner S, Grandl M, Liebisch G, Peer M, Orso E, Sigruner A, Sobota A, Schmitz G. oxLDL and eLDL Induced Membrane Microdomains in Human Macrophages. PLoS One. 2016;11:e0166798. doi: 10.1371/journal.pone.0166798 27870891 PMC5117723

[56] Kurose H. Cardiac Fibrosis and Fibroblasts. Cells. 2021;10. doi: 10.3390/cells10071716 34359886 PMC8306806

[57] Jiang Y, Cai R, Huang Y, Zhu L, Xiao L, Wang C, Wang L. Macrophages in organ fibrosis: from pathogenesis to therapeutic targets. Cell Death Discov. 2024;10:487. doi: 10.1038/s41420-024-02247-1 39632841 PMC11618518

[58] Shen S, Wang L, Liu Q, Wang X, Yuan Q, Zhao Y, Hu H, Ma L. Macrophage-to-myofibroblast transition and its role in cardiac fibrosis. Int Immunopharmacol. 2025;146:113873. doi: 10.1016/j.intimp.2024.113873 39693954

[59] Camici PG, d’Amati G, Rimoldi O. Coronary microvascular dysfunction: mechanisms and functional assessment. Nat Rev Cardiol. 2015;12:48–62. doi: 10.1038/nrcardio.2014.160 25311229

[60] Wang YT, Moura AK, Zuo R, Zhou W, Wang Z, Roudbari K, Hu JZ, Li PL, Zhang Y, Li X. Coronary Microvascular Dysfunction Is Associated With Augmented Lysosomal Signaling in Hypercholesterolemic Mice. J Am Heart Assoc. 2024;13:e037460. doi: 10.1161/JAHA.124.037460 39604023 PMC11681558

[61] Del Buono MG, Montone RA, Camilli M, Carbone S, Narula J, Lavie CJ, Niccoli G, Crea F. Coronary Microvascular Dysfunction Across the Spectrum of Cardiovascular Diseases: JACC State-of-the-Art Review. J Am Coll Cardiol. 2021;78:1352–1371. doi: 10.1016/j.jacc.2021.07.042 34556322 PMC8528638

[62] Paolisso P, Gallinoro E, Belmonte M, Bertolone DT, Bermpeis K, De Colle C, Shumkova M, Leone A, Caglioni S, Esposito G, Coronary Microvascular Dysfunction in Patients With Heart Failure: Characterization of Patterns in HFrEF Versus HFpEF. Circ Heart Fail. 2024;17:e010805. doi: 10.1161/CIRCHEARTFAILURE.123.010805 38108151

[63] Zhou B, Ren N, Geng J. Vericiguat improves cardiac function and microcirculation of a male patient with Fabry disease: A case report. Ann Noninvasive Electrocardiol. 2024;29:e13115. doi: 10.1111/anec.13115 38586938 PMC11000129

[64] Graziani F, Lillo R, Biagini E, Limongelli G, Autore C, Pieroni M, Lanzillo C, Calo L, Musumeci MB, Ingrasciotta G, Myocardial infarction with non-obstructive coronary arteries in hypertrophic cardiomyopathy vs Fabry disease. Int J Cardiol. 2022;369:29–32. doi: 10.1016/j.ijcard.2022.07.046 35931207

[65] Graziani F, Leccisotti L, Lillo R, Bruno I, Ingrasciotta G, Leone AM, Montone RA, Marano R, Rovere G, Indovina L, Coronary Microvascular Dysfunction Is Associated With a Worse Cardiac Phenotype in Patients With Fabry Disease. JACC Cardiovasc Imaging. 2022;15:1518–1520. doi: 10.1016/j.jcmg.2022.03.004 35926912

[66] Tomberli B, Cecchi F, Sciagra R, Berti V, Lisi F, Torricelli F, Morrone A, Castelli G, Yacoub MH, Olivotto I. Coronary microvascular dysfunction is an early feature of cardiac involvement in patients with Anderson-Fabry disease. Eur J Heart Fail. 2013;15:1363–1373. doi: 10.1093/eurjhf/hft104 23818648

[67] Bottillo I, Giordano C, Cerbelli B, D’Angelantonio D, Lipari M, Polidori T, Majore S, Bertini E, D’Amico A, Giannarelli D, A novel LAMP2 mutation associated with severe cardiac hypertrophy and microvascular remodeling in a female with Danon disease: a case report and literature review. Cardiovasc Pathol. 2016;25:423–431. doi: 10.1016/j.carpath.2016.07.005 27497751

[68] Lidove O, Belmatoug N, Froissart R, Lavigne C, Durieu I, Mazodier K, Serratrice C, Douillard C, Goizet C, Cathebras P, [Acid sphingomyelinase deficiency (Niemann-Pick disease type B) in adulthood: A retrospective multicentric study of 28 adult cases]. Rev Med Interne. 2017;38:291–299. doi: 10.1016/j.revmed.2016.10.387 27884455

[69] Fotoulaki M, Schuchman EH, Simonaro CM, Augoustides-Savvopoulou P, Michelakakis H, Panagopoulou P, Varlamis G, Nousia-Arvanitakis S. Acid sphingomyelinase-deficient Niemann-Pick disease: novel findings in a Greek child. J Inherit Metab Dis. 2007;30:986. doi: 10.1007/s10545-007-0557-3 17876723

[70] Hirota Y. A clinical study of left ventricular relaxation. Circulation. 1980;62:756–763. doi: 10.1161/01.cir.62.4.756 7190882

[71] Horinouchi K, Erlich S, Perl DP, Ferlinz K, Bisgaier CL, Sandhoff K, Desnick RJ, Stewart CL, Schuchman EH. Acid sphingomyelinase deficient mice: a model of types A and B Niemann-Pick disease. Nat Genet. 1995;10:288–293. doi: 10.1038/ng0795-288 7670466

[72] Hagemann N, Mohamud Yusuf A, Martiny C, Zhang X, Kleinschnitz C, Gunzer M, Kolesnick R, Gulbins E, Hermann DM. Homozygous Smpd1 deficiency aggravates brain ischemia/ reperfusion injury by mechanisms involving polymorphonuclear neutrophils, whereas heterozygous Smpd1 deficiency protects against mild focal cerebral ischemia. Basic Res Cardiol. 2020;115:64. doi: 10.1007/s00395-020-00823-x 33057972 PMC7560939

[73] Baechle JJ, Chen N, Makhijani P, Winer S, Furman D, Winer DA. Chronic inflammation and the hallmarks of aging. Mol Metab. 2023;74:101755. doi: 10.1016/j.molmet.2023.101755 37329949 PMC10359950

[74] Tan JX, Finkel T. Lysosomes in senescence and aging. EMBO Rep. 2023;24:e57265. doi: 10.15252/embr.202357265 37811693 PMC10626421

[75] Selman M, Pardo A. Fibroageing: An ageing pathological feature driven by dysregulated extracellular matrix-cell mechanobiology. Ageing Res Rev. 2021;70:101393. doi: 10.1016/j.arr.2021.101393 34139337

